# Bloodstream infection due to *Enterobacter ludwigii*, correlating with massive aggregation on the surface of a central venous catheter

**DOI:** 10.1007/s15010-020-01482-9

**Published:** 2020-09-03

**Authors:** Lysett Wagner, Frank Bloos, Slavena Vylkova

**Affiliations:** 1 Septomics Research Center, Leibniz Institute for Natural Product Research and Infection Biology – Hans Knöll Institute, Friedrich Schiller University, Albert-Einstein-Str. 10, 07745 Jena, Germany; 2 Center for Sepsis Control and Care (CSCC), Jena University Hospital, Jena, Germany; 3 Department of Anesthesiology and Intensive Care Therapy, University Hospital Jena, Jena, Germany

**Keywords:** *Enterobacter cloacae* complex, *Enterobacter ludwigii*, Catheter associated blood stream infection (CABSI), Sepsis

## Abstract

We report a case of catheter associated bloodstream infection due to *Enterobacter ludwigii* with a massive aggregation on the outside surface of a central venous catheter (CVC). The 57 years old patient with a history of spondylodiscitis and *Staphylococcus aureus*-associated endocarditis was admitted to the intensive care unit for acute cerebral infarction. The patient developed signs of infections and the CVC was removed 11 days after placement. The infectious agent was identified by standard diagnostics to the genus level as belonging to the *Enterobacter cloacae* complex, and additional molecular testing determined the species as *E. ludwigii*. The catheter was selected for a study aiming to identify the influence of blood components on the formation of central venous catheter-associated biofilms. In this course a massive biofilm was recognized and is presented here.

## Introduction

Catheter associated bloodstream infections (CABSI) are a common nosocomial infection acquired in intensive care units (ICU). Although CABSI are caused mainly by Gram-positive staphylococci and enterococci, infections with Gram-negative bacteria of the family Enterobacteriaceae like *Escherichia coli*, *Klebsiella* spp., or *Enterobacter* spp. are not uncommon [1, 2].

*Enterobacter* spp. are facultative aero-anaerobic motile bacteria that are constituent of the normal gut microbiota and frequently isolated from human-associated environments. Several species from this genus, such as *E. hormaechei* and *E. cloacae* [3], have been associated with nosocomial infections in immunocompromised patients. *E. cloacae* and other closely related species of the *Enterobacter cloacae* complex (ECC) are known to cause a range of life-threatening infections, including catheter-associated urinary tract infections and bacteremia [3].

According to the National Nosocomial Infections Surveillance system, 4.9% of the ICU acquired blood stream infections (BSI) were caused by *Enterobacter* spp. in the US between 1992 and 1999 [4]. Further, the annual epidemiological report “Healthcare-associated infections in intensive care units” for 2017 reports *Enterobacter* spp. as being responsible for 8.2% of the ICU CABSI cases in Europe [2].

Here we report a case of *E. ludwigii* caused CABSI correlating with a massive slimy aggregation on the outside of a central venous catheter (CVC), which was collected for a study investigating the influence of blood components on biofilm formation.

### Clinical case presentation

We report a 57 years old male patient who was treated in our ICU for an acute hemorrhagic cerebral infarction. The patient had a history of dorsal stabilization (Th 4/5–Th 9/10) after thoracic spine fracture complicated by *S. aureus* bacteremia. Four months later, the patient was re-admitted to the hospital with 39 °C fever and back pain. Diagnostic workup revealed a spondylodiscitis (Th10–Th11) and also a new endocarditis of the mitral valve with blood cultures positive for *S. aureus*. Antimicrobial therapy was initiated with flucloxacillin plus fosfomycin and the spine materials were removed. CVC placement into the right internal jugular vein occurred during preparation for surgery. At this point the patient was admitted to the ICU due to loss of vigilance requiring immediate sedation, endotracheal intubation and mechanical ventilation. The CT-scan revealed a hemorrhagic infarction in the area of the right medial artery with brain edema. This condition was treated by craniectomy. On the third day in ICU, we observed an increase in biomarkers of infection, but no fever—c-reactive protein increased from 137 to 246 mg/l, white blood cell count from 8.1 to 23 Gpt/l, and procalcitonin from 0.69 to 3.58 ng/ml. Blood cultures were obtained by peripheral puncture and via the central venous catheter. The CVC (day 11 after placement) was then removed and prepared for microbiological diagnostics and the study procedures. Blood cultures from all sites tested positive for *Enterobacter cloacae* complex. Antimicrobial therapy was escalated to ceftazidime, resulting in a timely decrease of the biomarkers of infection. Mitral valve vegetation was no longer visible in subsequent echocardiography. The patient was referred to a rehabilitation clinic after 28 days of ICU therapy where he was released with good neurological outcome. Written informed consent for study participation was obtained from the patient’s legal representative; the study was approved by the ethics committee of the Jena University Hospital.

### Microbiology

**Fig. 1:**
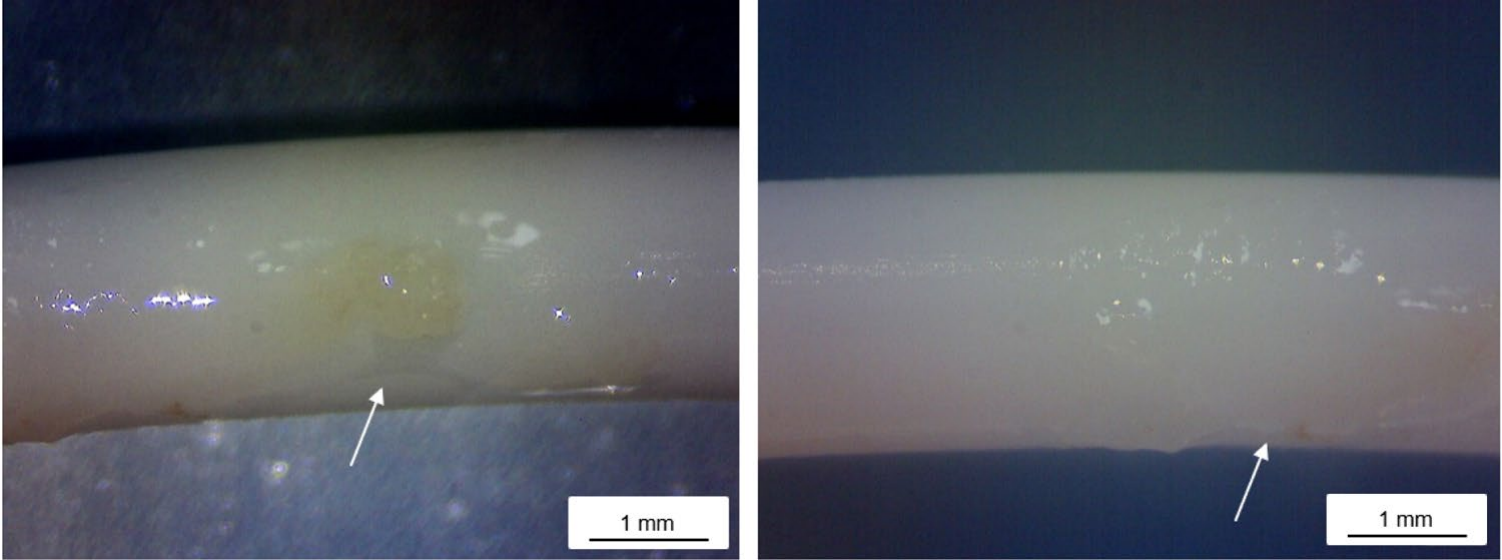
Central venous catheter surface with aggregations**.** Immediately after removal the CVC was transferred in sterile phosphate buffered saline and placed at 4 °C. On the next day a prominent slimy aggregation, about 8 cm apart from the catheter tip, was noted. Arrows point to the border of the aggregation

Next to the routine diagnostic microbiology, the catheter was examined in the context of a study aiming to explore the influence of blood components adherent to CVC on microbial biofilm formation. Sterile handling of the CVC was practiced throughout. The CVC was removed from the patient’s chest, stored in PBS buffer containing protease inhibitor (complete™ ULTRA Tablets, Merck, Germany), and stored at 4 °C overnight. After removal of the PBS, a prominent aggregation, visible by the bare eye, was detected on the outside of the catheter around 8 cm apart from the tip. Using a stereomicroscope (Stemi508, Carl Zeiss Microscopy, Germany) a spatial definable, white to yellowish colored, slimy film covering an area of about 7 mm^2^ became evident (Fig. 1). A fraction of this film was scraped using a sterile scalpel and sampled on CNA agar with 5% sheep blood as well as on tryptic soy agar. The plates were inoculated at 37 °C. After 8 h grey and slimy colonies without hemolytic activity were observed on CNA. On TSB agar the colonies were beige and slimy. To determine species identity, colony PCRs was performed in order to sequence the 16S rDNA (704F: 5´-GTAGCGGTGAAATGCGTAGA, 1495R: 5´-CTACGGCTACCTTGTTACGA [5]). The 16S sequence analysis showed 100% sequence identity with *E. ludwigii* type strain EN-119 (NCBI Accession CPO17279.1) using the NCBI BLASTn tool applying the reference dataset. Based on this result, *Enterobacter* spp. established genes (hsp60, primer adapted from [6]: Hsp60-F: 5´-GGTAGAAGAAGGCGTRGTHGC, Hsp60-R: 5´-ATGCAYTCGGTVGTGATCATCAG; rpoB, primer adapted from [7]: rpoB-f: 5´-AAAAACGTATTCGTAAGGATTTTGGTAA, rpoB-r2: 5´-CCAGCAGATCCAGGCTCAGCTCCATGTT) were amplified and sequenced in a second step to assure species identity. Both sequences confirmed the identification of the strain as *E. ludwigii* with 100% sequence identity to type strain sequences.

Sequences are deposited on GenBank (Accession numbers: MT299750 (16S rDNA), MT276843 (hsp60), MT276844 (rpoB)) and the strain (JMRC:ST:036,472) is accessible at the Jena Microbial Resource Collection (JMRC, Jena, Germany).

## Discussion

The clinical standard diagnostic proved a blood stream infection and the involvement of the catheter in this infection due to positive central line and peripheral blood culture, as well as pathogen detection on the CVC tip. In parallel, due to the integration of the catheter in a translational study investigating the role of blood components on CVC-associated biofilm formation, the aggregation on the outer side of the catheters was recognized and the pathogen was independently isolated. Consistently, the agent was identified as a member of the ECC. Sequencing of additional marker assigned the pathogen to *E. ludwigii*.

In general, *E. ludwigii* strains are intrinsically resistant to ampicillin, amoxicillin, first-generation cephalosporins and to cefoxitin due to the production of a beta-lactamase [3]. Some strains exhibit resistance to third generation cephalosporine, but not to cotrimoxazole, gentamicin, imipenem, and ciprofloxacin [3]. Furthermore, genes mediating carbapenem resistance [8] and genes mediating extended spectrum β-lactamases (ESBLs) [9] were described in *E. ludwigii*. In our case, the agent was susceptible to meropenem, a carbapenem, and to ceftazidime, a third generation cephalosporine. Biomarkers of infection decreased when the patient was treated with ceftazidime.

The pathogen *E. ludwigii* belonging to the ECC was described in 2005 based on 16 isolates of different clinical samples (urinary tract, respiratory tract, infected and uninfected skin, blood, or stool) [10]. Only a few clinical cases were described to this date. *Enterobacter ludwigii* was isolated from orthopedic implants infections [11], a surgical wound [8], BSIs in neonates [9], and CABSI in hemodialysis patients [12, 13]. Additionally, 18 *E. ludwigii* isolates were reported in a single study mainly from blood cultures, genitourinary and gastrointestinal tract [14].

The true incidence of ECC species remains concealed due to a lack in accuracy in routine diagnostics. The standard clinical diagnostics apply either physiological approaches (e.g., Api 20E gallery, Vitek 2) or MALDI-TOF approaches. With respect to the EEC, these systems can identify the species complex, but not differentiate the diversity within the ECC [3]. Next to the standard, molecular as well as physiological methods can differentiate *E. ludwigii* from the other members of the ECC [10].

*Enterobacter* ssp. are known to form biofilms and infections are often associated with indwelling medical devices like catheters, which are known spots of microbial biofilm formation. The few *E. ludwigii* cases known from the literature are associated with orthopedic implants, urinary catheters, hemodialysis catheters and CVCs, which assumes a role of biofilm formation. Although almost nothing is known about the ability of *E. ludwigii* to form biofilm, it can be assumed to be similar as described for other species in the ECC. The presence of mobility mediating flagella which facilitates adhesion and protein export, both relevant in biofilm formation [3], is such example. Interestingly, the level of biofilm formation of *E. cloacae* is strain dependent [15]. An important aspect of biofilms is the reduced effectiveness of antibiotic treatment most likely due to physical barrier and reduced metabolic activity of cells in the biofilm. These resting pathogens in the biofilm together with the potentials in antibiotic resistance, make *E. ludwigii* and the other opportunistic species of the ECC a severe issue in ICUs.

Retrospective studies in clinical specimen collections might reveal *E. ludwigii* as a more frequent agent of nosocomial infection. A prospectively study of 196 clinical ECC strains found 9% *E. ludwigii *isolates [14]. Based on the molecular phylogeny it should be re-evaluated if a (sub-) typing of the clades in the ECC with MALDI-TOF is possible. Therefore, a more precise diagnostic can help to identify virulence traits and epidemiological aspects of the species within the ECC. Biofilm assays of all ECC group’s isolates could define species dependent behavior regarding biofilm formation.

Currently, it is not clear if the infection with *E. ludwigii* can lead to massive biofilm aggregations on catheters. We report this to clinicians and the scientific community to heighten the awareness for a potential reservoir in such biofilms. Microbes embedded in a biofilm are significantly less susceptible to antibiotic treatment and it might be necessary to remove the infected medical device to eliminate the infection [16].

## Conclusion

With this report we contribute a case of catheter associated bloodstream infection by a non-frequent opportunistic pathogen forming massive aggregate on the outside of a CVC. The strain isolated from this catheter was identified as *E. ludwigii*, a rapidly growing, biofilm-forming species. The isolated strain was made accessible to the scientific community in order to activate further research into the diagnostic, as well as to promote studies on the virulence traits of *E. ludwigii* and the ECC.
